# No-Reference Quality Assessment Method for Blurriness of SEM Micrographs with Multiple Texture

**DOI:** 10.1155/2019/4271761

**Published:** 2019-06-02

**Authors:** Hui Wang, Xiaojuan Hu, Hui Xu, Shiyin Li, Zhaolin Lu

**Affiliations:** ^1^School of Information and Control Engineering, China University of Mining and Technology, Xuzhou, China; ^2^School of Physics, China University of Mining and Technology, Xuzhou, China; ^3^School of Computer Science and Technology, China University of Mining and Technology, Xuzhou, China; ^4^Advanced Analysis and Computation Centre, China University of Mining and Technology, Xuzhou, China

## Abstract

Scanning electron microscopy (SEM) plays an important role in the intuitive understanding of microstructures because it can provide ultrahigh magnification. Tens or hundreds of images are regularly generated and saved during a typical microscopy imaging process. Given the subjectivity of a microscopist's focusing operation, blurriness is an important distortion that debases the quality of micrographs. The selection of high-quality micrographs using subjective methods is expensive and time-consuming. This study proposes a new no-reference quality assessment method for evaluating the blurriness of SEM micrographs. The human visual system is more sensitive to the distortions of cartoon components than to those of redundant textured components according to the Gestalt perception psychology and the entropy masking property. Micrographs are initially decomposed into cartoon and textured components. Then, the spectral and spatial sharpness maps of the cartoon components are extracted. One metric is calculated by combining the spatial and spectral sharpness maps of the cartoon components. The other metric is calculated on the basis of the edge of the maximum local variation map of the cartoon components. Finally, the two metrics are combined as the final metric. The objective scores generated using this method exhibit high correlation and consistency with the subjective scores.

## 1. Introduction

Scanning electron microscopy (SEM) helps researchers intuitively understand microstructures because it can provide ultrahigh magnification. SEM is playing an increasingly important role in various research areas, such as medical imaging, automated inspection, bioimaging, and ore detection. At present, microscopists must deal with a considerable number of images because tens or hundreds of images are regularly generated and saved during a typical microscopy imaging process [1–3]. Images obtained via SEM may be blurred because of the imaging equipment used or the operators who performed the process. Frequently, only a few images are useful for further analysis. Given the subjectivity of the SEM operation, blurring is a major distortion in SEM images [4–6]. Postek and Vladár qualitatively and quantitatively analysed the sharpness of micrographs in the Fourier domain [7, 8]. Their work provided valuable information to the SEM community. In addition, their research was easy to understand and the meaning was clear. However, Postek and Vladár disregarded the characteristics of the human visual system (HVS), which is the final receiver of the images. Thus, HVS characteristics should be considered.

Image quality assessment (IQA) is a useful method for finding clear images [9–11]. Human beings are the ultimate receivers of processed images, and they judge image quality. Automatic approaches that can consistently assess the image quality with human subjective evaluation must be developed [10]. Subjective IQA methods require numerous observers to participate in experiments. These methods are not only expensive and time-consuming but they also cannot be incorporated into automatic or real-time image systems [9]. Hence, objective quality methods that can automatically and accurately assess image quality must be developed. Objective IQA methods can be classified into three categories on the basis of the availability of the reference images: full reference (FR) [9, 10], in which complete reference images are required; reduced reference (RR) [12, 13], in which partial information about the reference images is available; and no reference (NR) [14–16], in which no information about the reference images is necessary. Given that NR images are available, the use of the FR and RR methods is limited in microscopy applications.

NR assessment methods for blurriness or sharpness can be divided into three categories [17]. (1) Edge-based approaches assume that image edges will spread when blurriness occurs. Marziliano et al. detected vertical edges by using the Sobel operator and then obtained a blurriness measure from the edge map. The average width of all edge pixels was regarded as a metric for blurriness [18]. Ferzli and Karam combined Weber's law with Marziliano's method and proposed the “just noticeable blur” method, which considers HVS properties [14]. (2) Transform-based approaches: blurriness leads to distortion in different domains, and thus, many methods assess blurriness on the basis of transform domains, such as discrete cosine transform [19], discrete wavelet transform (DWT) [20], and sparse transform [21]. Some methods utilise information from two or more domains. Chen and Bovik used the comprehensive spatial and gradient information of DWT as the final blurriness metric [16]. Vu et al. combined spatial features with spectral features as a sharpness measurement [22]. Li et al. proposed a robust method by learning multiscale features extracted in the spatial and spectral domains [23]. (3) Statistical pixel information-based approaches analyse the distribution laws of the pixels of original images or their maps. Considering that blurriness decreases the variance of difference in the intensity of adjacent pixels, Tsomko and Kim calculated the block difference variance and regarded it as a blurriness metric [24]. Bahrami and Kot developed a method based on the maximum local variation (MLV) distribution of each pixel, and the standard deviation of distribution was used to measure sharpness [25]. Li et al. combined the sum of squared non-DC moment (SSM) values of a gradient map that were computed on the basis of their Tchebichef moments, block variances, and visual saliency to measure blurriness [26]. Edge-based methods rely excessively on the image content. If an image only has few sharp edges, then edge-based methods may lead to inaccuracy. Transform-based methods assume that distortion in certain domains can be easily extracted and computed; however, these methods occasionally overlook human visual perception, which plays a key role in assessment. Statistical pixel information-based methods are not robust because they are sensitive to noise.

**Figure 1:**
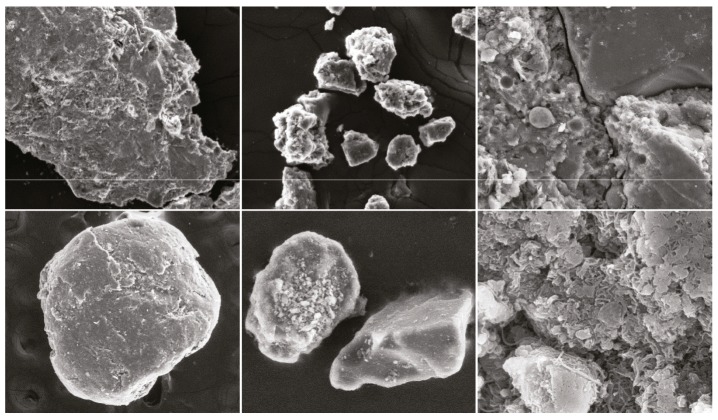
Several samples in the micrograph database.

Samples should be preprocessed before SEM imaging. This procedure leads to the difference between micrographs and natural images. Samples are typically ores, which are polished and placed on conductive tapes. Therefore, the micrograph content evidently has an edge area.  Figure 1 shows that the final micrographs have many textures. Micrographs used in the database have more strong edges and textures than natural images. Images can be decomposed into cartoon and textured components due to the entropy masking property of human visual perception [27]. The cartoon components of images contain strong edges and flat areas, whereas the textured components contain middle- and high-frequency information, including noise and textures. In [28], Attneave indicated that image information is concentrated on contours. Certain areas and objects are described simply by HVS on the basis of the Gestalt perception psychology [29]. Moreover, HVS is more sensitive to changes in cartoon components than to changes in textured components.

The experiments in the succeeding sections also prove that the cartoon components exhibit better blurriness features than the original micrographs. This study proposes a new NR assessment method that initially decomposes SEM micrographs into cartoon and textured components. The blurriness of the cartoon components is then assessed. The assessment method based on the combination of spatial-spectral information and spatial map edges is adopted to separately calculate two different metrics and to obtain the final metric via the weighted summation of the two metrics after normalization. The experiments demonstrate the good performance of the proposed method.

## 2. Cartoon+Textured Components with Isotropic Nonlinear Filters

Before assessing the quality of the SEM micrographs, the micrographs are initially decomposed into cartoon and textured components. An original SEM micrograph is denoted as *f*, which can be decomposed into the cartoon component *u* and textured component *v*. The decomposition process is defined as *f* = *u* + *v*. The general variational framework for the decomposition model is provided in Meyer's models [30] as an energy minimisation problem:

(1) $$\begin{array}{c} \underset{\left(u,v\right)\in X_{1}\times X_{2}}{\inf}\left\{F_{1}\left(u\right)+\lambda F_{2}\left(v\right)\text{: }f=u+v\right\}, \end{array}$$

where *F*_1_, *F*_2_ ≥ 0 are functions and *X*_1_ and *X*_2_ are spaces of functions or distributions. *F*_1_(*u*) < ∞ and *F*_2_(*v*) < ∞ if and only if (*u*, *v*) ∈ *X*_1_ × *X*_2_. *λ* > 0 is a tuning parameter. The cartoon component contains the strong edges and low-frequency information of a micrograph, which can be described as *F*_1_(*u*) ≪ *F*_2_(*u*). By contrast, the textured component contains the noise and texture of a micrograph, which can be described as *F*_1_(*v*) ≫ *F*_2_(*v*).

A fast and approximate solution for the general variational problem was proposed in [31, 32] by applying a nonlinear low-pass/high-pass filter pair. For each point *x* of a micrograph, when the micrograph is filtered with a low-pass filter and if *x* is a cartoon component, the total variation (TV) does not decrease. By contrast, if *x* is a textured component, then the TV decreases rapidly. A nonlinear filter was proposed by the solution on the basis of this characteristic. The local total variation (LTV) at *x* is defined as follows:

(2) $$\begin{array}{c} {\text{LTV}}_{\sigma }\left(x\right)=L_{\sigma }*\left|\text{Df}\left(x\right)\right|, \end{array}$$

where *L*_*σ*_ is a low-pass filter with a Gaussian kernel and a standard deviation of *σ*, $\text{Df}\left(x\right)=\int_{\Omega }\sqrt{{\left(f_{x}\right)}^{2}+{\left(f_{y}\right)}^{2}}dx\;dy$, Ω denotes the entire micrograph region, and ∗ is a convolution symbol.

*λ*_*σ*_ is the relative reduction rate of the LTV, which is defined as follows:

(3) $$\begin{array}{c} \lambda_{\sigma }=\frac{{\text{LTV}}_{\sigma }\left(x\right)-{\text{LTV}}_{\sigma }\left(L_{\sigma }*x\right)}{{\text{LTV}}_{\sigma }\left(x\right)}. \end{array}$$

This formula denotes the decrease rate of *x*'s LTV when *x* is filtered using a low-pass filter *L*_*σ*_. If *λ*_*σ*_ is close to 0, then the LTV is slightly reduced and pixel *x* belongs to the cartoon region. If *λ*_*σ*_ is close to 1, then the LTV is large and *x* belongs to the textured region. The proposed fast nonlinear low-pass and high-pass filter pair is defined as follows:

(4) $$\begin{array}{c} u\left(x\right)=w\left(\lambda_{\sigma }\left(x\right)\right)\left(L_{\sigma }*x\right)+\left(1-w\left(\lambda_{\sigma }\left(x\right)\right)\right)x,\\ v\left(x\right)=f\left(x\right)-u\left(x\right), \end{array}$$

where *w*(*α*) is the soft threshold function. The function is defined as follows:

(5) $$\begin{array}{c} w\left(\alpha \right)=\left\{\begin{array}{cc} 0, & \alpha \leq a_{1},\\ \frac{\alpha -a_{1}}{a_{2}-a_{1}}, & a_{1}\leq \alpha \leq a_{2},\\ 1, & \alpha \geq a_{2}, \end{array}\right. \end{array}$$

and its parameters *a*_1_ and *a*_2_ are fixed in the experiments. The specific numbers are *a*_1_ = 0.25 and *a*_2_ = 0.5 [32].

**Figure 2:**
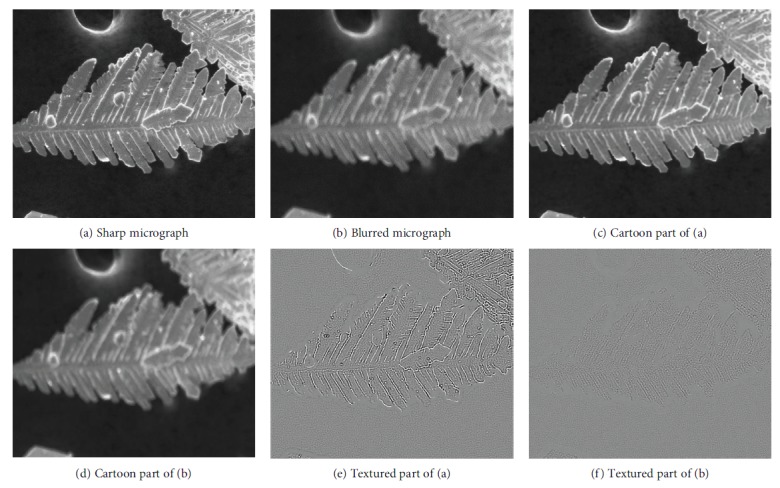
Sharp and blurred micrographs and their cartoon and textural parts.

The attenuation of the high-frequency content is caused by the blurriness of an image. Therefore, if an image is more blurred, then its cartoon component is more similar to that of the original image.  Figure 2 compares between the cartoon and textured decompositions of micrographs with different blurriness extent but the same content. The parameter *σ* of the nonlinear filter is 3. Compared with that of the sharp micrograph, the cartoon component of the blurred micrograph looks more similar to that of the original micrograph because in the blurred micrograph, more of the pixels' *λ*_*σ*_ are close to 0. Thus, more regions of the micrograph belong to the cartoon component. Therefore, the textured part of the blurred micrograph is less evident than that of the sharp micrograph.

**Figure 3:**
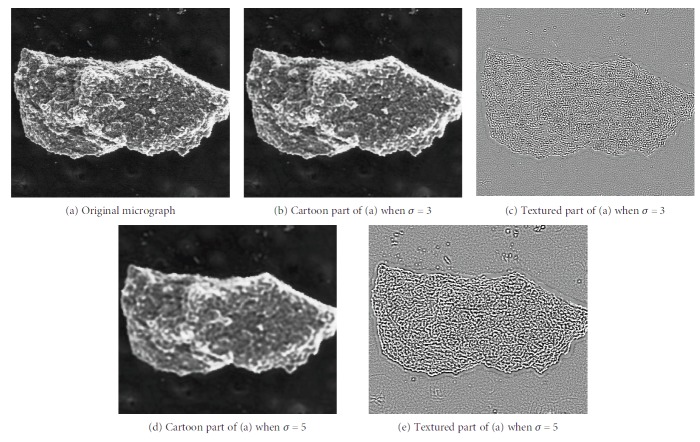
Cartoon and textural parts under different decomposition parameters.

Figure 3 presents the different decomposition results of various parameter *σ* values. Compared with *σ* = 3, more regions of the original micrograph belong to the textured component when *σ* = 5. Although more noises and textures are separated, the edges of the cartoon component exert greater zigzag effect. If *σ* is small, then the cartoon component still contains texture. If *σ* is large, then some edges are regarded as texture. A zigzag effect also occurs on the cartoon component.

In this study, *σ* = 3. The cartoon component is a simplified description of the original micrograph, and it contains strong edges that are perceived more easily by human visual perception when blurriness occurs. As previously mentioned, human visual perception is sensitive to distortion in edges. The cartoon component retains the original edges, simplifies the original micrograph, and does not lose distortion information. Although the textured component is also affected by blurriness, human visual perception is less sensitive to distortion in this part because of perceptual redundancy. Furthermore, noise and repetitive texture reduce the performance of assessment methods, as proven in the following sections. The experiments also prove that assessing the quality of the cartoon component is better than that of the original micrograph.

## 3. NR IQA Method for Blurriness

Distortion at the edges should be given attention in accordance with the characteristics of human visual perception; thus, this method is primarily edge-based. Apart from strong edges, the cartoon component also contains other frequency information that is utilised to measure the attenuation of high-frequency information caused by blurriness. Attenuation is measured using a transform-based approach. The final metric is a weighted summation of the edge- and transform-based metrics.

**Figure 4:**
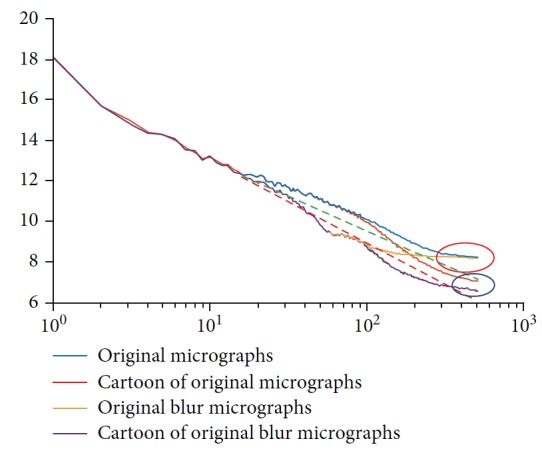
Log-log spectrum of Figure 2.

A famous property called the 1/*f* law exists in the spectrum domain. This property describes the amplitude spectrum of an image as an approximately straight line on a log-log scale [33, 34]. If blurriness appears in images, then the absolute value of the line's slope increases, particularly in the high-frequency content [35]. For original images, if the tails of curves overlap (red mark in Figure 4), then the accuracy of quality assessment decreases. The curves of the cartoon components retain the trend caused by blurriness and avoid overlapping tails (blue mark in Figure 4). The blue solid line in Figure 4 is a log-log spectrum curve of the image in Figure 2(a), whereas the yellow line belongs to the image in Figure 2(b). The brown line is the image in Figure 2(c), and the purple curve is the image in Figure 2(d). The green dashed line is the fitted straight line of the brown curve at high frequencies, whereas the red dashed line is the fitted straight line of the purple curve at high frequencies.

The spectral map generated using the method mentioned in the spectral and spatial sharpness algorithm [22] intuitively proves that the cartoon component plays a more important role than its original micrograph in quality assessment. The spectral map is defined as *S*_1_ and is calculated using the previously mentioned slope. The absolute value of the slope is defined as *α*. To obtain *α*, the algorithm initially calculates the 2D discrete Fourier transform *y*_*γ*_(*f*, *θ*) of micrograph *γ*. In *y*_*γ*_(*f*, *θ*), *f* and *θ* are computed using the following:

(6) $$\begin{array}{c} f={\left[{\left(\frac{u}{m/2}\right)}^{2}+{\left(\frac{v}{m/2}\right)}^{2}\right]}^{1/2},\\ \theta =\arctan\left(\frac{v}{u}\right), \end{array}$$

where *u* ∈ [−*m*/2, *m*/2] and *v* ∈ [−*m*/2, *m*/2]. *z*_*γ*_(*f*) is the summed magnitude spectrum, as given by

(7) $$\begin{array}{c} z_{\gamma }\left(f\right)=\underset{\theta }{\sum }\left|y_{\gamma }\left(f,\theta \right)\right|. \end{array}$$

**Figure 5:**
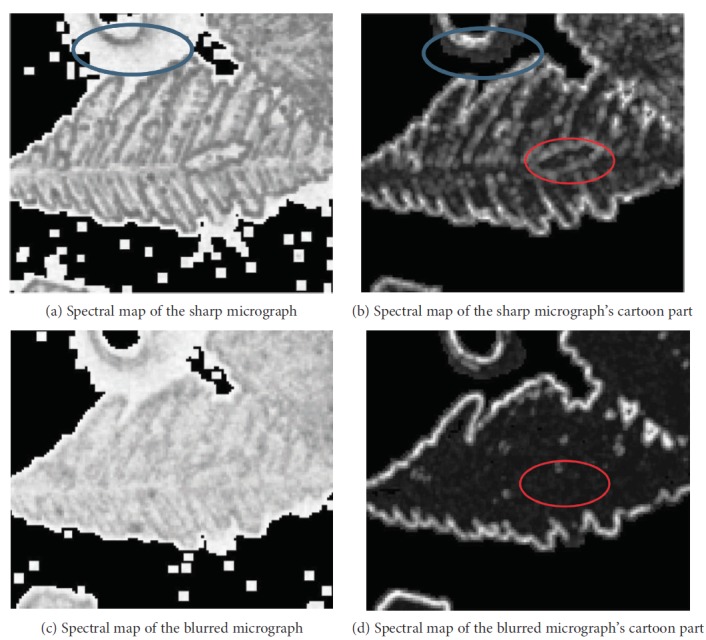
Spectral maps of micrographs and their cartoon parts generated by S3.

The algorithm finds a line that best fits the magnitude spectrum. *α* is the absolute value of the slope of the line. *α* is calculated by

(8) $$\begin{array}{c} \alpha_{\min}=\arg\underset{\alpha }{\min}{\left\|\beta f^{-\alpha }-z_{\gamma }\left(f\right)\right\|}_{2}^{2}, \end{array}$$

where *L*_2_ − norm is taken over all radial frequency *f* > 0. Finally, *S*_1_ is defined by

(9) $$\begin{array}{c} S_{1}=1-\frac{1}{1+e^{\tau_{1}\left(\alpha_{\min}-\tau_{2}\right)}}, \end{array}$$

where *τ*_1_ = −3 and *τ*_2_ = 2. For additional details, refer to [22].  Figure 5 shows that the spectral map of the cartoon component contains no noise and efficiently distinguishes high-low-frequency content. By contrast, the spectral map of the original micrograph misconstrues some low-frequency content as high-frequency content (marked by the blue ellipses). The blurriness of high-frequency content still exists (marked by the red ellipses), thereby indicating that the blurriness in the cartoon component conforms well to HVS.

The cartoon component extracts blurriness features well in the spectral and spatial domains. The spatial maps in spectral and spatial sharpness (S3) [22] and the MLV [25] are generated using the local variation of an image. In S3, map *S*_2_ is generated on the basis of the TV. The TV of micrograph *γ* is defined as *v*_*γ*_, and

(10) $$\begin{array}{c} v_{\gamma }=\frac{1}{255}\underset{i,j}{\sum }\left\{\left|I_{i,\text{j}}-I_{x,y}\right|x=i-1,i,i+1,y=j-1,j,j+1\right\}, \end{array}$$

where *I*_*x*,*y*_(*i* − 1 ≤ *x* ≤ *i* + 1, *j* − 1 ≤ *y* ≤ *j* + 1) are eight-neighbour pixels of *I*_*i*,*j*_. *S*_2_ is computed using

(11) $$\begin{array}{c} S_{2}\left(\gamma \right)=\frac{1}{4}\underset{\xi \in \gamma }{\max}v\left(\xi \right), \end{array}$$

where *ξ* is a 2 × 2 block of *γ*. The final *S*_3_(*γ*) map is defined as follows:

(12) $$\begin{array}{c} S_{3}\left(\gamma \right)=S_{1}{\left(\gamma \right)}^{\mu }\times {\text{S}}_{3}{\left(\gamma \right)}^{1-\mu }, \end{array}$$

where 0 ≤ *μ* ≤ 1; *μ* was set as 0.5 in [22]. For the reducing effect of the noise, the average sharpness is 1% of the highest values of *S*_3_(*γ*). Additional details are provided in [22]. The MLV also generates its map. The MLV is defined as follows:

(13) $$\begin{array}{c} \varphi \left(I_{i,j}\right)=\left\{\max\left|I_{i,\text{j}}-I_{x,y}\right|x=i-1,i,i+1,y=j-1,j,j+1\right\}, \end{array}$$

where *I*_*x*,*y*_(*i* − 1 ≤ *x* ≤ *i* + 1, *j* − 1 ≤ *y* ≤ *j* + 1) are eight neighbours of *I*_*i*,*j*_. Given a micrograph with size *M* × *N*, the MLV is calculated for each pixel *I*_*i*,*j*_ at location (*i*, *j*) using formula (13). The final MLV map is generated via

(14) $$\begin{array}{c} \psi \left(I\right)=\left(\begin{array}{ccc} \varphi \left(I_{1,1}\right) & \cdots & \varphi \left(I_{1,N}\right)\\ \vdots & \ddots & \vdots \\ \varphi \left(I_{M,1}\right) & \cdots & \varphi \left(I_{M,N}\right) \end{array}\right). \end{array}$$

**Figure 6:**
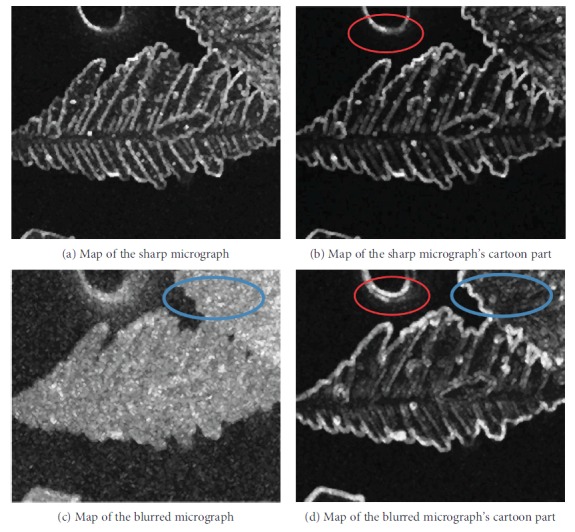
Spatial maps of micrographs and their cartoon parts generated by S3.

**Figure 7:**
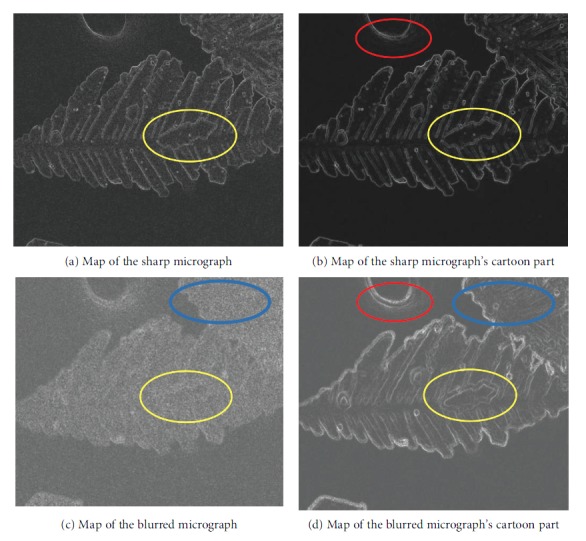
Spatial maps of micrographs and their cartoon parts generated by the MLV.

Additional details are available in [25]. Given the separation of texture, the spatial map of the cartoon components focuses on edges. Figures 6 and 7 provide the intuitive details. In Figure 6, the region marked by the blue ellipse in the blurred micrograph shows that the textures and edges are mixed and measuring distortions at the edges is difficult. In its cartoon component, we observe that more pixels are considered edges. The red-marked region indicates that the cartoon component still contains distortions caused by blurriness. The same condition appears in Figure 7. The yellow-marked region confirms that the MLV can extract blur distortions better than the sum of the local variations because of the clear edges of the MLV map.

**Figure 8:**
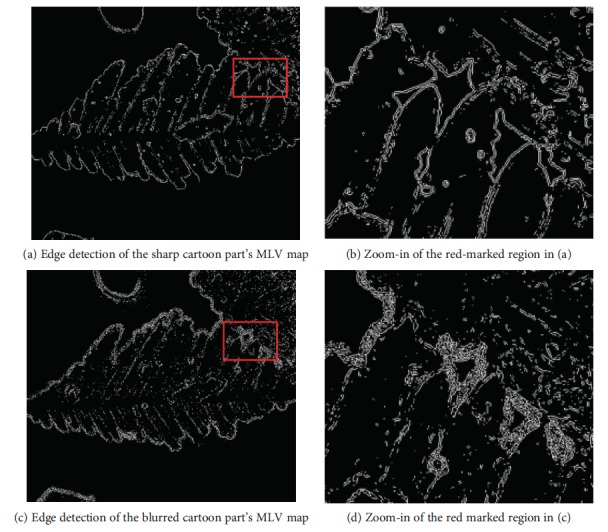
Edge detections of the MLV maps and their zoom-in.

As we all know, blurriness leads to the spread of edges.  Figure 8 shows the edge of the cartoon component's MLV spatial map. From the details of the micrographs in Figures 7(a) and (7)c, we observe that the edge detection of the blurry MLV map has more edge pixels than that of the sharp one and its edge widths are wider than those of the sharp one.

In [25], the textured component and edges exhibit high MLV, thereby indicating that high variations in pixel intensities are better indicators of sharpness than low variations. The cartoon component has edges and blank content, but blurriness does not change the blank content. Thus, we do not utilise the statistics of the MLV distribution as [25] did. In this research, we detect edges of the MLV map and calculate the sparsity of edge pixels as a blurriness metric. We define the sparsity of edge pixels as the average distance of pixels at edges. For pixels that correspond to an edge location, the start and end positions of the edge are defined as the location of the local luminance extrema closest to the edge. Edge width is defined as the length between the start and end positions [18]. The final blur metric sparsity is generated by the following:

(15) $$\begin{array}{c} \text{Sparsity}=\frac{\text{Sum of all edgewidths}}{\text{Number of edges}}. \end{array}$$

Blurriness causes the spread of edges. It also produces more edge pixels during edge detection. The experiments indicate that the sparsity of edge pixels is lower when the micrograph is more blurred.

**Figure 9:**
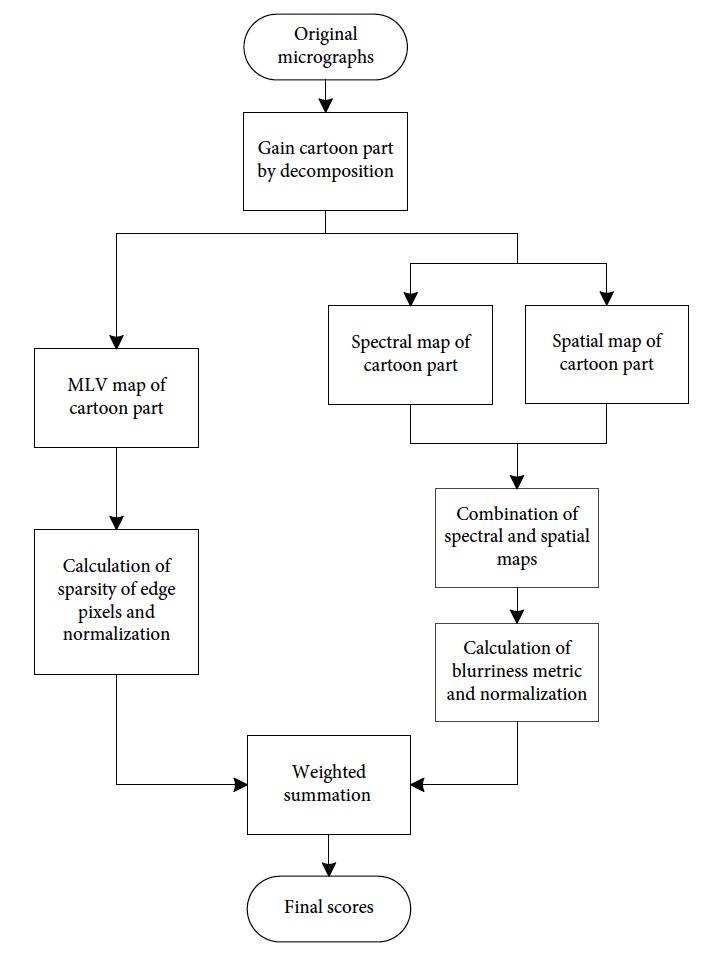
Flow chart of the proposed method.

On the basis of the preceding analysis, this study proposes a new assessment method. The flowchart of this method is presented in Figure 9. An original micrograph is initially decomposed into cartoon and textured components. Then, the spectral and spatial features with the sparsity of edge pixels are combined. The final score is obtained via weighting summation. The method extracts spectral and spatial features using the algorithm in S3, and the metric is defined as *S*. We separately calculate the sparsity of edge pixels in the vertical and horizontal directions. Vertical sparsity is *d*_*v*_, whereas horizontal sparsity is *d*_*h*_. The final sparsity *d* is defined as

(16) $$\begin{array}{c} d=\sqrt{d_{v}^{2}+d_{h}^{2}}, \end{array}$$

and the final score *Q* is obtained using

(17) $$\begin{array}{c} Q=\frac{S*\eta }{\max\left(\text{S}\right)}+\frac{d*\left(1-\eta \right)}{\max\left(d\right)}, \end{array}$$

where *η* is a weighting coefficient.

## 4. Analysis and Discussion of Experiment Results

### 4.1. SEM Micrographs and Their Quality Assessment Results

**Figure 10:**
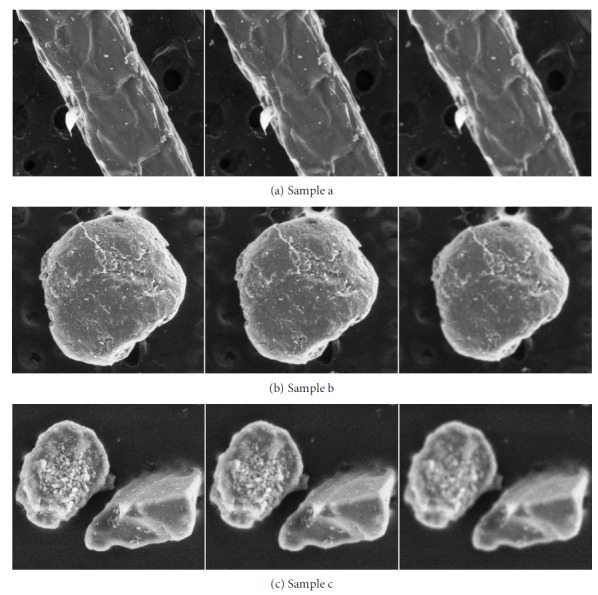
Three samples and their micrographs with different blurriness extent.

The SEM micrographs used in this research were taken at the Modern Analysis and Computing Centre of China University of Mining and Technology. We selected 50 samples. For every sample, we obtained three blurred micrographs with different extent by artificially adjusting the SEM focus parameter. After obtaining 150 micrographs, 30 SEM users without knowledge in image processing participated in the subjective experiment. Every micrograph gained 30 scores. To reduce the error of the experimental results, we selected 30 scores based on confidence interval and eliminated 5 scores that were not found in the confidence interval. The final mean opinion score (MOS) was the average score of the remaining 25 scores. Apart from the two samples presented in Figures 1 and 2, three other samples are illustrated in Figure 10.

The blurriness extent increases from the first column to the last column. In Table 1, the higher the blurriness extent, the more blurred the micrograph is. The subjective and objective assessment scores are also provided in Table 1. MOS is the mean opinion score, *S* is the objective score obtained from the combination of spectral and spatial features, *d* is the sparsity of edge pixels, and *Q* is the final objective score. With regard to these parameters, the lower their values, the more blurred the micrograph is. This analysis matches the one mentioned in Section 3.

### 4.2. Performance Analysis of the Proposed Objective Method

In this study, three performance indexes are adopted to measure the proposed objective method.

(1) Pearson Linear Correlation Coefficient (PLCC):
  (18) $$\begin{array}{c} \text{PLCC}=\frac{1}{n-1}\overset{n}{\underset{i=1}{\sum }}\left(\frac{x_{i}-\bar{x}}{\sigma_{x}}\right)\left(\frac{y_{i}-\bar{y}}{\sigma_{y}}\right), \end{array}$$
  where {*x*_1_, *x*_2,_⋯, *x*_*n*_} are subjective scores, {*y*_1_, *y*_2,_⋯, *y*_*n*_} are objective scores, $\bar{x}$ and $\bar{y}$ are their average scores, and *σ*_*x*_ and *σ*_*y*_ are their variances. PLCC is a metric that measures how well the objective scores correlate with the subjective scores. If PLCC is higher, then the correlation is better.
(2) Root-Mean-Square Error (RMSE):
  (19) $$\begin{array}{c} \text{RMSE}={\left[\frac{1}{n}\overset{n}{\underset{i=1}{\sum }}{\left(x_{i}-y_{i}\right)}^{2}\right]}^{1/2}. \end{array}$$
  RMSE is a metric that measures the absolute error between the subjective and objective scores. A good algorithm is supposed to have a low RMSE value.
(3) Spearman's Rank Ordered Correlation Coefficient (SROCC):
  (20) $$\begin{array}{c} \text{SROCC}=1-\frac{6}{n\left(n^{2}-1\right)}\overset{n}{\underset{i=1}{\sum }}{\left(r_{x_{i}}-r_{y_{i}}\right)}^{2}, \end{array}$$
  where *r*_*x*_*i*__ and *r*_*y*_*i*__ are the rank positions of *x*_*i*_ and *y*_*i*_ in arrays {*x*} and {*y*}, respectively. SROCC is a metric that measures the relative monotonicity between the subjective and objective scores. A high SROCC value indicates a good algorithm.

**Figure 11:**
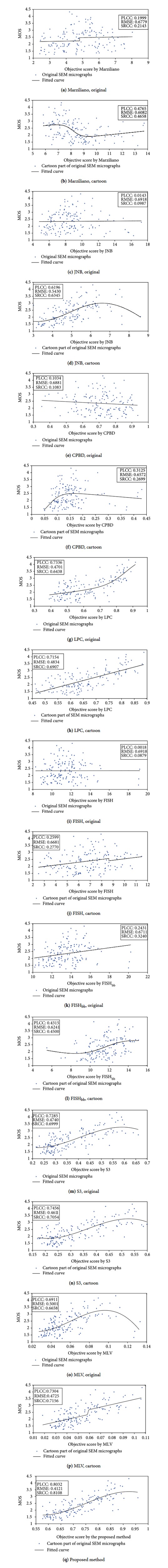
Fitted curves by different methods.

Figure 11 presents eight pairs of comparison between the original micrographs and their cartoon components. We obtain three performance indexes and fitted curves of the subjective and objective scores using eight different methods [14, 18, 22, 25, 36–38]. The fitted curves of the cartoon component are evidently better than those of the original micrographs. The performance indexes validate this finding. Therefore, the distortion in the cartoon components of the micrographs conforms more to the observed distortion by HVS when blurriness occurs.

**Figure 12:**
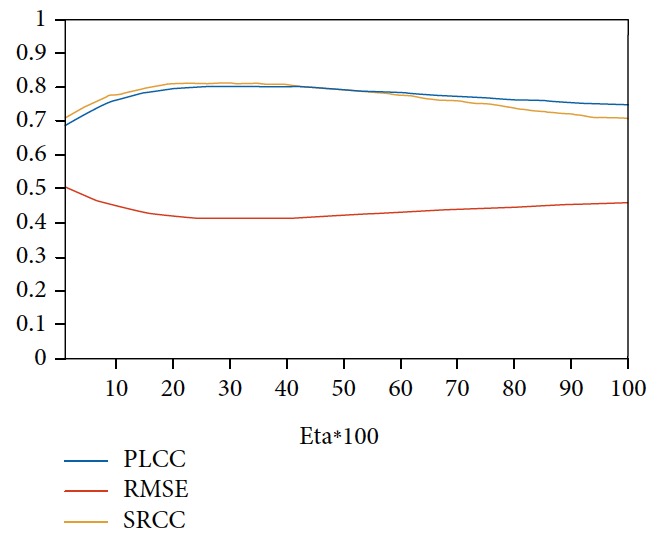
Performance indexes with different *η*.

The last graph in Figure 11 shows the fitted curve of the subjective and objective scores obtained using the proposed method, and three performance indexes are appended at the top left corner.  Figure 12 presents the analysis of *η*. The values of PLCC, RMSE, and SRCC are the best when *η* = 0.3. Therefore, in the proposed method, the weighting coefficient is assumed as 0.3. This also indicates HVS is more sensitive to blurred distortion at the edges.  Table 2 provides a summary of the performance indexes generated using different methods.

The top two indexes are marked in boldface. We obtain two conclusions from Table 2. (1) Cartoon components reflect blurriness characteristics better than original micrographs. (2) The indexes of the proposed method are the best compared with those of the other eight methods. Thus, the proposed method is the most similar to HVS perception characteristics.

## 5. Conclusion

This study proposes a new method for evaluating the blurriness of SEM micrographs. HVS is more sensitive to the distortion of cartoon components than that of redundant texture components according to the Gestalt perception psychology and the entropy masking property. The method initially decomposes original micrographs into cartoon and textured components. Then, blurriness features are extracted from the cartoon components. When assessing the quality of the cartoon components, the method combines the micrographs' spectral-spatial features and the sparsity of edge pixels of the MLV spatial map. Finally, we obtain the final quality scores via the weighted summation of the two metrics. The experiments demonstrate that the proposed method is more similar to human visual perception than other state-of-art methods when assessing the quality of SEM micrographs.

## Figures and Tables

**Table 1:** Assessment result of the different extent of blurriness.

Sample	Extent of blurriness	MOS	*Q*	*S*	*d*
*a*	1	3.5417	0.8536	0.4889	5.1535
	2	2.84	0.7207	0.2425	4.9760
	3	1.9167	0.6653	0.1893	4.7207
*b*	1	3.125	0.8404	0.4877	4.5334
	2	2.542	0.7603	0.3025	4.5111
	3	2.08	0.6775	0.1855	4.2694
*c*	1	2.5	0.7738	0.3339	4.5139
	2	1.625	0.6708	0.2211	4.1157
	3	1.542	0.6071	0.1957	3.7384

**Table 2:** Summary of the performance indexes generated by different methods.

Methods	Micrographs	PLCC	RMSE	SROCC
Marziliano	Original	0.1999	0.6779	0.2143
	Cartoon parts	0.4765	0.6082	0.4658
JNB	Original	0.0143	0.6918	0.0987
	Cartoon parts	0.6196	0.543	0.6345
CPBD	Original	0.1034	0.6881	0.1083
	Cartoon parts	0.3125	0.6572	0.2699
LPC	Original	0.7336	0.4701	0.6438
	Cartoon parts	0.7154	0.4834	0.6907
FISH	Original	0.0018	0.6918	0.0879
	Cartoon parts	0.2599	0.6681	0.277
FISH_bb_	Original	0.2431	0.6711	0.324
	Cartoon parts	0.4315	0.6241	0.45
S3	Original	0.7285	0.474	0.6999
	Cartoon parts	**0.7456**	**0.4611**	0.7054
MLV	Original	0.6911	0.5001	0.6658
	Cartoon parts	0.7304	0.4725	**0.7156**
Proposed		**0.8032**	**0.4121**	**0.8108**

## Data Availability

The data including the database of blurred micrographs, MOS (mean opinion scores), and objective scores used to support the findings of this study are available from the corresponding author upon request.

## References

[1] Koho S., Fazeli E., Eriksson J. E., Hänninen P. E. (2016). Image quality ranking method for microscopy. *Scientific Reports*.

[2] Zotta M. D., Han Y., Bergkoetter M. D., Lifshin E. (2016). An evaluation of image quality metrics for scanning electron microscopy. *Microscopy and Microanalysis*.

[3] Zeder M., Kohler E., Pernthaler J. (2010). Automated quality assessment of autonomously acquired microscopic images of fluorescently stained bacteria. *Cytometry Part A*.

[4] Firestone L., Cook K., Culp K., Talsania N., Preston K. (1991). Comparison of autofocus methods for automated microscopy. *Cytometry*.

[5] Brenner J. F., Dew B. S., Horton J. B., King T., Neurath P. W., Selles W. D. (1976). An automated microscope for cytologic research a preliminary evaluation. *Journal of Histochemistry & Cytochemistry*.

[6] Ellenberger S. L. (2000). *Influence of Defocus on Measurements in Microscope Images, [M.S. thesis]*.

[7] Chae Postek M. L. T., Vladár A. E. (1998). Image sharpness measurement in scanning electron microscopy—part I. *Scanning*.

[8] Vladár A. E., Postek M. T., Davidson M. P. (1998). Image sharpness measurement in scanning electron microscopy—part II. *Scanning*.

[9] Wang Z., Bovik A. C., Sheikh H. R., Simoncelli E. P. (2004). Image quality assessment: from error visibility to structural similarity. *IEEE Transactions on Image Processing*.

[10] Sheikh H. R., Bovik A. C., De Veciana G. (2005). An information fidelity criterion for image quality assessment using natural scene statistics. *IEEE Transactions on Image Processing*.

[11] Sheikh H. R., Bovik A. C. (2006). Image information and visual quality. *IEEE Transactions on Image Processing*.

[12] Wang Z., Bovik A. C. (2011). Reduced- and no-reference image quality assessment. *IEEE Signal Processing Magazine*.

[13] Soundararajan R., Bovik A. C. (2012). RRED indices: reduced reference entropic differencing for image quality assessment. *IEEE Transactions on Image Processing*.

[14] Ferzli R., Karam L. J. (2009). A no-reference objective image sharpness metric based on the notion of just noticeable blur (JNB). *IEEE Transactions on Image Processing*.

[15] Ye P., Kumar J., Kang L., Doermann D. Unsupervised feature learning framework for no-reference image quality assessment.

[16] Chen M.-J., Bovik A. C. (2011). No-reference image blur assessment using multiscale gradient. *EURASIP Journal on Image and Video Processing*.

[17] Wang Z.-M. (2015). Review of no-reference image quality assessment. *Acta Automatica Sinica*.

[18] Marziliano P., Dufaux F., Winkler S., Ebrahimi T. (2004). Perceptual blur and ringing metrics: application to JPEG2000. *Signal Processing: Image Communication*.

[19] Saad M. A., Bovik A. C., Charrier C. (2012). Blind image quality assessment: a natural scene statistics approach in the DCT domain. *IEEE Transactions on Image Processing*.

[20] Ferzli R., Karam L. J. No-reference objective wavelet based noise immune image sharpness metric.

[21] Li L., Wu D., Wu J., Li H., Lin W., Kot A. C. (2016). Image sharpness assessment by sparse representation. *IEEE Transactions on Multimedia*.

[22] Vu C. T., Phan T. D., Chandler D. M. (2012). S3: a spectral and spatial measure of local perceived sharpness in natural images. *IEEE Transactions on Image Processing*.

[23] Li L., Xia W., Lin W., Fang Y., Wang S. (2017). No-reference and robust image sharpness evaluation based on multi-scale spatial and spectral features. *IEEE Transactions on Multimedia*.

[24] Tsomko E., Kim H. J. Efficient method of detecting globally blurry or sharp images.

[25] Bahrami K., Kot A. C. (2014). A fast approach for no-reference image sharpness assessment based on maximum local variation. *IEEE Signal Processing Letters*.

[26] Li L., Lin W., Wang X., Yang G., Bahrami K., Kot A. C. (2016). No-reference image blur assessment based on discrete orthogonal moments. *IEEE Transactions on Cybernetics*.

[27] Watson A. B., Borthwick R., Taylor M. Image quality and entropy masking.

[28] Attneave F. (1954). Some informational aspects of visual perception. *Psychological Review*.

[29] Koffka K. (2013). *Principles of Gestalt Psychology*.

[30] Meyer Y. (2001). *Oscillating Patterns in Image Processing and Nonlinear Evolution Equations: the Fifteenth Dean Jacqueline B. Lewis Memorial Lectures*.

[31] Buades A., le T. M., Morel J. M., Vese L. A. (2010). Fast cartoon + texture image filters. *IEEE Transactions on Image Processing*.

[32] Buades A., Lisani J. L. (2016). Directional filters for cartoon + texture image decomposition. *Image Processing On Line*.

[33] Ruderman D. L. (1994). The statistics of natural images. *Network: Computation in Neural Systems*.

[34] Srivastava A., Lee A. B., Simoncelli E. P., Zhu S. C. (2003). On advances in statistical modeling of natural images. *Journal of Mathematical Imaging and Vision*.

[35] Field D. J., Brady N. (1997). Visual sensitivity, blur and the sources of variability in the amplitude spectra of natural scenes. *Vision Research*.

[36] Narvekar N. D., Karam L. J. (2011). A no-reference image blur metric based on the cumulative probability of blur detection (CPBD). *IEEE Transactions on Image Processing*.

[37] Hassen R., Wang Z., Salama M. M. A. (2013). Image sharpness assessment based on local phase coherence. *IEEE Transactions on Image Processing*.

[38] Vu P. V., Chandler D. M. (2012). A fast wavelet-based algorithm for global and local image sharpness estimation. *IEEE Signal Processing Letters*.

